# Therapeutic activation of G protein-coupled estrogen receptor 1 in Waldenström Macroglobulinemia

**DOI:** 10.1186/s40164-022-00305-x

**Published:** 2022-09-12

**Authors:** Eugenio Morelli, Zachary R. Hunter, Mariateresa Fulciniti, Annamaria Gullà, Ida Daniela Perrotta, Valeria Zuccalà, Cinzia Federico, Giada Juli, Martina Manzoni, Domenica Ronchetti, Enrica Romeo, Maria Eugenia Gallo Cantafio, Debora Soncini, Lorenza Maltese, Marco Rossi, Aldo M. Roccaro, Michele Cea, Pierfrancesco Tassone, Antonino Neri, Steven C. Treon, Nikhil C. Munshi, Giuseppe Viglietto, Nicola Amodio

**Affiliations:** 1 Department of Medical Oncology, Jerome Lipper Multiple Myeloma Center, Dana-Farber Cancer Institute, Boston, MA 02215 USA; 2 Harvard Medical School, Boston, MA 02215 USA; 3 Laboratory of Transmission Electron Microscopy, Department of Biology, Ecology and Earth Sciences, Centre for Microscopy and Microanalysis, University of Calabria, Cosenza, Italy; 4 Pathology Unit, “Pugliese-Ciaccio” Hospital, 88100 Catanzaro, Italy; 5 Department of Experimental and Clinical Medicine, Magna Graecia University, 88100 Catanzaro, Italy; 6 Clinical Research Development and Phase I Unit, ASST Spedali Civili Di Brescia, Brescia, Italy; 7 Department of Hematology, Fondazione Cà Granda IRCCS Policlinico, 20122 Milan, Italy; 8 Clinic of Hematology, Department of Internal Medicine (DiMI), University of Genoa, Genoa, Italy; 9 IRCCS Ospedale Policlinico San Martino, Genoa, Italy; 10 Scientific Directorate, Azienda USL-IRCCS Reggio Emilia, 42123 Reggio Emilia, Italy; 11 VA Boston Healthcare System, Boston, MA 02132 USA

## Abstract

Activating G protein-coupled estrogen receptor 1 (GPER1) is an attractive therapeutic strategy for treating a variety of human diseases including cancer. Here, we show that GPER1 is significantly upregulated in tumor cells from different cohorts of Waldenström Macroglobulinemia (WM) patients compared to normal B cells. Using the clinically applicable GPER1-selective small-molecule agonist G-1 (also named Tespria), we found that pharmacological activation of GPER1 leads to G2/M cell cycle arrest and apoptosis both in vitro and in vivo in animal models, even in the context of the protective bone marrow *milieu*. Activation of GPER1 triggered the TP53 pathway, which remains actionable during WM progression. Thus, this study identifies a novel therapeutic target in WM and paves the way for the clinical development of the GPER1 agonist G-1.

**Supplementary Information:**

The online version contains supplementary material available at 10.1186/s40164-022-00305-x.

To the Editor,

Waldenström Macroglobulinemia (WM) is a rare hematologic malignancy characterized by the accumulation of IgM-secreting lymphoplasmacytic lymphoma cells within a permissive bone marrow microenvironment [1]. Activating mutations in MYD88 are present in 93–97% of WM patients [1], while the tumor suppressor TP53 remains unaffected in most patients and thus susceptible to intervention [2]. Only a few WM patients achieve complete remission with the current standard-of-care treatments, highlighting the need for novel therapies.

G protein-coupled estrogen receptor 1 (GPER1) is a membrane estrogen receptor that regulates cell growth, migration, apoptotic cell death, and other cancer-related biological functions [1, 3–7]. Its pharmacological activation by the selective small-molecule agonist G-1 or its enantiomer LNS8801 is emerging as an attractive therapeutic strategy in human malignancies [5–8], as increasing GPER1 activity frequently increases p53 expression [9]. Therefore, we investigated GPER1 and its pharmacologic activation in WM.

## GPER1 is upregulated in WM

**Fig. 1:**
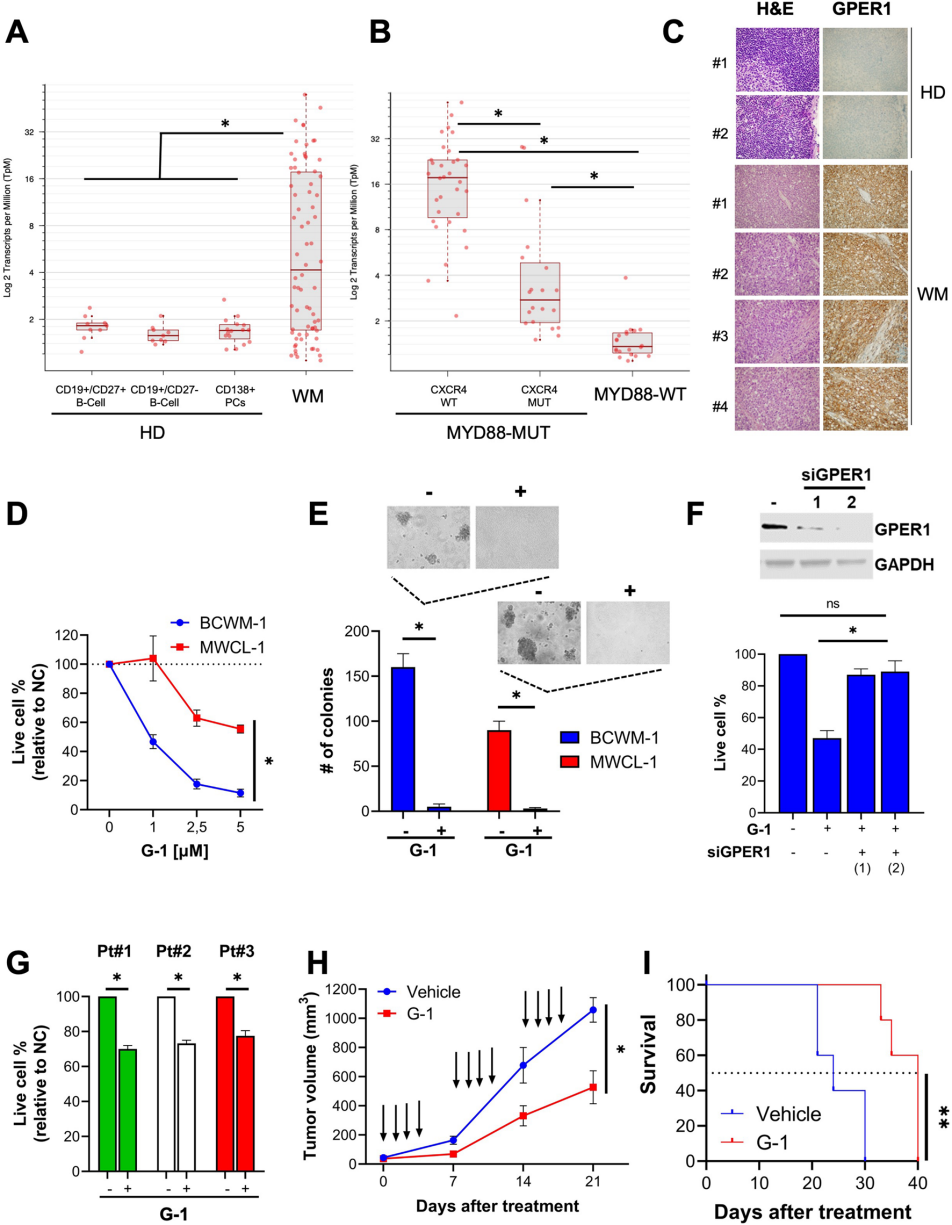
GPER1 is upregulated in WM, and its pharmacological activation triggers in vitro and in vivo anti-tumor activity. **A** RNA-seq analysis of GPER1 mRNA expression in CD19 + cells from WM patients (*n* = 72) and healthy donor (HD)-derived B cells. These latter included CD19 + /CD27 + B cells (*n* = 9), CD19 + /CD27 + B cells (*n* = 9) and CD138 + plasma cells (*n* = 16). **B** RNA-seq analysis of GPER1 mRNA expression in WM patients carrying MYD88-WT (*n* = 20) or MYD88-MUT (*n* = 52), with the latter further divided into CXCR4-WT (*n* = 32) or CXCR4-MUT (*n* = 20). **C** IHC analysis of GPER1 performed in lymph node specimens from WM patients (*n* = 4) and normal lymph nodes (*n* = 2), (20× magnification). **D** Cell viability was assessed by CTG assay 72 h after treatment with G-1 at the indicated doses. **E** A colony assay was performed in methylcellulose using WM cells treated with 1 μM G-1 for 72 h; representative pictures of colonies (10× magnification) are reported. **F** BCWM-1 cells were transfected with [100 nM] scrambled siRNAs or two different GPER1-targeting siRNAs (siGPER1#1 and siGPER#2), and then treated with G-1 [1 μM] or DMSO. GPER expression and cell viability were assessed 48 h after transfection by WB and CTG respectively. **G** Cell viability was assessed in primary CD19 + cells by CTG assay 72 h after treatment with G-1 [1 μM] at the indicated doses. **H** Average and SD of tumor volume (mm3) from groups of mice (*n* = 5/group) versus time (days) when tumor was measured. BCMW.1 cells (5 × 106 in 100 mL of serum-free RPMI1640 medium) were implanted in the flank of NOD/SCID mice. After tumor detection, mice were randomized to intraperitoneal treatment with G-1 [1 mg/Kg] or vehicle. Data are mean tumor volume ± SD. Arrows represent treatments. **I** Kaplan–Meier survival plot showing survival for mice treated with vehicle or G-1. *p < 0.05 from a Wilcoxon rank sum test in **A**, from a pairwise comparison using Wilcoxon rank sum exact test in **B**, and from Student’s t-test in other panels. **p < 0.05 from a log-rank test

We used RNA-seq to analyze the expression of *GPER1* mRNA in CD19^+^ cells from WM patients (*n* = 72) and in healthy donor–derived B cells. These latter included CD19^+^/CD27^+^ B cells (*n* = 9), CD19^+^/CD27^+^ B cells (*n* = 9) and CD138^+^ plasma cells (*n* = 16). We found a remarkable upregulation of *GPER1* in WM (Fig. 1A). Moreover, by analyzing clinically relevant patient subgroups [1], we observed higher *GPER1* expression in patients carrying activating mutations in MYD88 and a wild-type *CXCR4* gene (Fig. 1B). We further validated *GPER1* mRNA upregulation by querying the GSE9656 and GSE61597 datasets of WM patients (Additional file 1: Fig. S1A, B). Moreover, we confirmed the upregulation of GPER1 protein expression by IHC analysis of lymph node samples derived from WM patients compared to healthy donors (Fig. 1C). Finally, we showed that BCWM-1 and MWCL-1 WM cell lines express GPER1 mRNA and protein similarly to the positive control breast cancer cell line MCF7(Additional file 1: Fig. S2A, B).

## Pharmacological activation of GPER1 antagonizes tumor cell growth in WM, both in vitro and in vivo

We next studied the effect of GPER1 pharmacological manipulation using selective agonist (G-1) and antagonist (G-36 and G-15) [10]. We found that low-micromolar G-1 concentrations reduced the growth (Fig. 1D) and clonogenicity (Fig. 1E) of BCWM-1 and MWCL-1 WM cell lines. These effects were abrogated after genetic silencing of GPER1, confirming on-target activity (Fig. 1F). G-1 antagonized the growth of CD19^+^ cells from three WM patients (Fig. 1G) while sparing B cells from healthy donors (Additional file 1: Fig. S2C). A treatment cycle with G-1 resulted in a significant reduction of tumor growth in a clinically relevant BCWM-1 xenograft model (Fig. 1H), and prolonged animal survival (Fig. 1I). On the other hand, GPER1 antagonists G-36 and G-15 promoted the survival of BCWM-1 and MWCL-1 cells (Additional file 1: Fig. S2D).

## Pharmacological activation of GPER1 triggers the TP53 pathway in WM

**Fig. 2:**
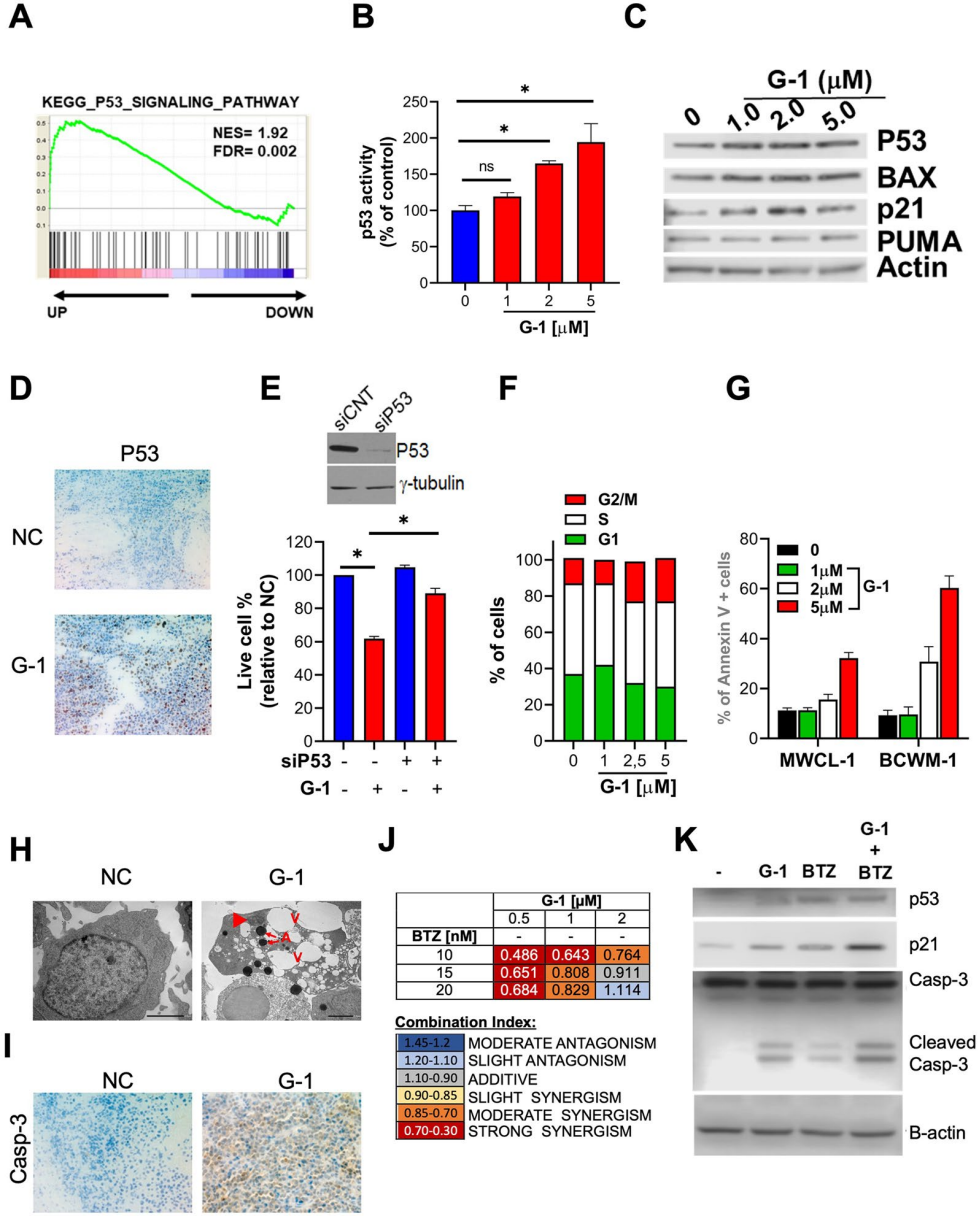
GPER1 pharmacological activation inhibits cell cycle progression and triggers apoptosis via inducing the p53 pathway. **A** GSEA performed 48 h after treatment with 1 μM G-1. **B** A p53 luminometric reporter assay was used to evaluate p53 transcriptional activity in G-1-treated BCWM-1 cells. **C** WB analysis of p53, p21, BAX, and PUMA in primary CD19^+^ WM cells treated with G-1 for 24 h. **D** Immunohistochemical staining for p53 (×20) in tumors sectioned on day 21 from vehicle- or G-1 [1 mg/kg] treated mice. Photographs are representative of one mouse receiving each treatment. **E** BCWM-1 cells were transfected with scrambled siRNAs (siCNT) or p53 targeting siRNAs and, after 24 h, were treated with vehicle or 1 μM G-1 for an additional 24 h and assessed for cell viability by CTG assay. WB analysis reports p53 knock-down in siP53-transfected cells. **F** FACS analysis of cell cycle phases of BCWM-1 cells 24 h after treatment with vehicle or G-1. **G** Annexin V staining of BCWM-1 cells 24 h after treatment with vehicle or G-1. **H** TEM analysis of BCWM-1 cells treated with G-1 (1 μM) or DMSO (NC). Control cells appear well-preserved with intact mitochondria, orderly chromatin folding and a clear nuclear membrane. Apoptotic cells become pyknotic with many electron-transparent vacuoles (V), chromatin (arrowhead) and cytoplasm condensation (increase in electron density of cytoplasmic matrix and organelles) and formation of apoptotic bodies (**A**). **I** Immunohistochemical staining for caspase 3 (×20) in tumors sectioned on day 21 from vehicle- or G-1 [1 mg/kg] treated mice. **J** Table showing combination indexes resulting from combinatorial treatments of BCWM-1 with G-1 and bortezomib (24 h time point). **K** WB analysis of p53, p21 and caspase 3 in BCWM-1 cells treated with [0.5 μM] G-1 and 10 nM bortezomib (BZ)

In WM cells treated with G-1, a gene set enrichment analysis (GSEA) found activation of the TP53 (p53) pathway (Fig. 2A), which was further confirmed using a reporter assay measuring p53 transcriptional activity (Fig. 2B). Consistently, G-1 increased the protein expression of p53 and its targets p21, BAX, BAD, and PUMA in BCWM-1 cells (Additional file 1: Fig. S2A) and CD19^+^ cells from a WM patient (Fig. 2C). Increased p53 protein expression was also observed in tumors retrieved from a SCID/NOD mouse treated with G-1 (Fig. 2D). Importantly, genetic silencing of p53 significantly antagonized the growth inhibitory effects of G-1 in BCWM-1 cells (Fig. 2E).

## Pharmacological activation of GPER1 induces cell cycle arrest and apoptosis in WM

G-1 promoted a dose-dependent accumulation of BCWM-1 cells in the G2/M phase (Fig. 2F), with a concomitant increase in the expression of mitotic protein cyclin B1 (Additional file 1: Fig. S3B). G-1 also increased annexin V binding (Fig. 2G) and caspase 3/7 activity (Additional file 1: Fig. S3C), which are markers of apoptotic cell death. Apoptosis was further confirmed by WB analysis of cleaved PARP, caspase 3 and 7 (Additional file 1: Fig. S3D), and by transmission electron microscopy (TEM) revealing the appearance of typical apoptotic features (Fig. 2H). IHC analysis highlighted an increase in the expression of caspase 3 in BCWM-1 xenografts retrieved from mice treated with G-1 (Fig. 2I). The anti-WM activity of G-1 was maintained even in the presence of protective bone marrow stromal cells (Additional file 1: Fig. S3E). Combining G-1 with the proteasome inhibitor bortezomib, a clinically active compound that activates p53 in tumor cells [11, 12], led to synergistic anti-proliferative activity (Fig. 2J), along with a synergistic activation of p53 target p21 and cleaved caspase 3 (Fig. 2K).

## Conclusion

This study shows GPER1 is a novel actionable target in WM, providing the framework for translation of G-1 to clinical trials.

## Supplementary Information

**Additional file 1: Figure S1.** Analysis of GPER1 mRNA in public datasets GSE9656 (A) and GSE61597 (B). GSE9656: we analyzed GPER1 mRNA in CD19-selected peripheral blood B cells (pBCs; n = 7), bone marrow B cells from WM (WM-BCs; n = 12), bone marrow plasma cells from healthy donors (BM-PCs; n = 10), and WM plasma cells (WM-PCs, n = 9). GSE61597: we analyzed GPER1 mRNA in normal bone marrow CD25+ (n = 7) and CD25– (n = 9) B cells, clonal B cells from newly diagnosed patients with IgM MGUS (n=22), smoldering (n = 17), and symptomatic WM (n = 10). **Figure S2.** A. qRT-PCR analysis of GPER1 mRNA in MCF7 breast cancer cell line (positive control), MWCL-1 and BCWM-1 WM cell lines, and CD19+ primary cells from four WM patients. B. WB analysis of GPER1 protein in a panel of six cancer cell lines (MCF7, MWCL-1, BCWM-1, DAUDI, RAJI, and MEC1). GAPDH was used as a loading control. C. CTG viability assay in BCWM-1 cells treated with indicated concentrations of GPER1 antagonists G15 and G-36. *Indicates p < 0.05 from a Student’s t-test. Ns indicates p > 0.05 from a Student’s t-test. **Figure S3.** A. WB analysis of p53, p21, BAX, and PUMA in primary CD19+ WM cells treated with G1 for 24 h. B. Wb analysis of Cyclin B1 in WM cell lines treated with indicated concentrations of G1. GAPDH was used as a loading control. C. Caspase 3/7 activity assay in WM cell lines treated with the indicated concentrations of G-1. Activity is represented relative to untreated cells. D. WB analysis of PARP, cleaved PARP, Caspase-3, and cleaved Caspase-3 in WM cell lines treated with the indicated concentrations of G-1. E. CTG viability assay and Caspase 3/7 activity assay in BCWM-1 cells, co-cultured for 48h with patient-derived bone marrow stromal cells and treated with G-1 [1 µM] or control. *Indicates p < 0.05 from a Student’s t-test.

**Additional file 2. **Methods.

## Data Availability

The authors declare that all data supporting the findings of this study are available within the article and its Additional Information. Files or reagents are available from the corresponding authors on request.
