# Supplementary material for: Therapeutic activation of G protein-coupled estrogen receptor 1 in Waldenström Macroglobulinemia
Source: Exp Hematol Oncol. 2022 Sep 12;11:54. doi: 10.1186/s40164-022-00305-x (PMC9469525; doi:10.1186/s40164-022-00305-x)
Supplement: Supplementary file 1 — Additional file 1: Figure S1. Analysis of GPER1 mRNA in public datasets GSE9656 (A) and GSE61597 (B). GSE9656: we analyzed GPER1 mRNA in CD19-selected peripheral blood B cells (pBCs; n = 7), bone marrow B cells from WM (WM-BCs; n = 12), bone marrow plasma cells from healthy donors (BM-PCs; n = 10), and WM plasma cells (WM-PCs, n = 9). GSE61597: we analyzed GPER1 mRNA in normal bone marrow CD25+ (n = 7) and CD25– (n = 9) B cells, clonal B cells from newly diagnosed patients with IgM MGUS (n=22), smoldering (n = 17), and symptomatic WM (n = 10). Figure S2. A. qRT-PCR analysis of GPER1 mRNA in MCF7 breast cancer cell line (positive control), MWCL-1 and BCWM-1 WM cell lines, and CD19+ primary cells from four WM patients. B. WB analysis of GPER1 protein in a panel of six cancer cell lines (MCF7, MWCL-1, BCWM-1, DAUDI, RAJI, and MEC1). GAPDH was used as a loading control. C. CTG viability assay in BCWM-1 cells treated with indicated concentrations of GPER1 antagonists G15 and G-36. *Indicates p < 0.05 from a Student’s t-test. Ns indicates p > 0.05 from a Student’s t-test. Figure S3. A. WB analysis of p53, p21, BAX, and PUMA in primary CD19+ WM cells treated with G1 for 24 h. B. Wb analysis of Cyclin B1 in WM cell lines treated with indicated concentrations of G1. GAPDH was used as a loading control. C. Caspase 3/7 activity assay in WM cell lines treated with the indicated concentrations of G-1. Activity is represented relative to untreated cells. D. WB analysis of PARP, cleaved PARP, Caspase-3, and cleaved Caspase-3 in WM cell lines treated with the indicated concentrations of G-1. E. CTG viability assay and Caspase 3/7 activity assay in BCWM-1 cells, co-cultured for 48h with patient-derived bone marrow stromal cells and treated with G-1 [1 µM] or control. *Indicates p < 0.05 from a Student’s t-test. [file 40164_2022_305_MOESM1_ESM.pptx]

## Slide 1
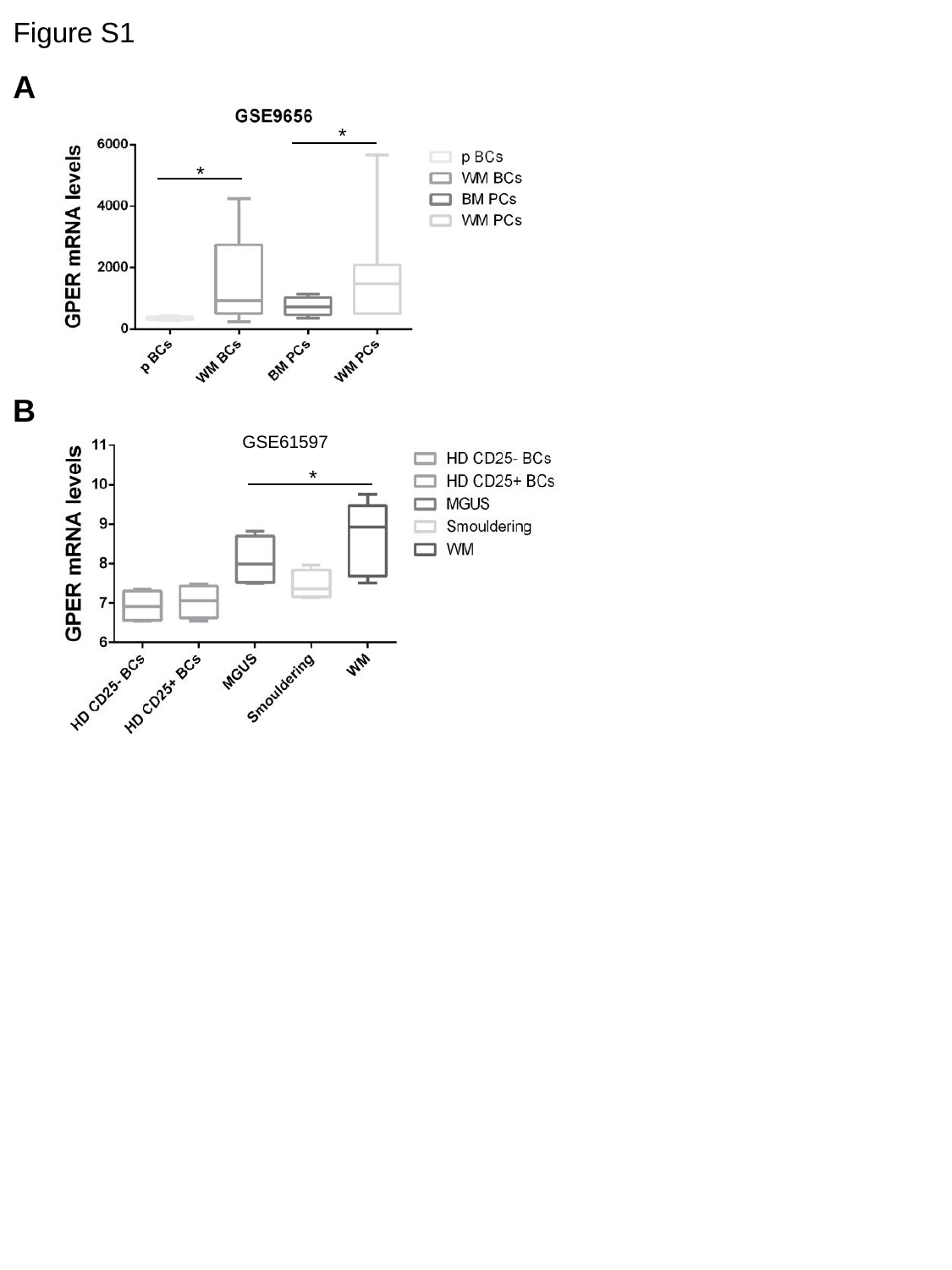

Figure S1
A
*
*
B
GSE61597
*

## Slide 2
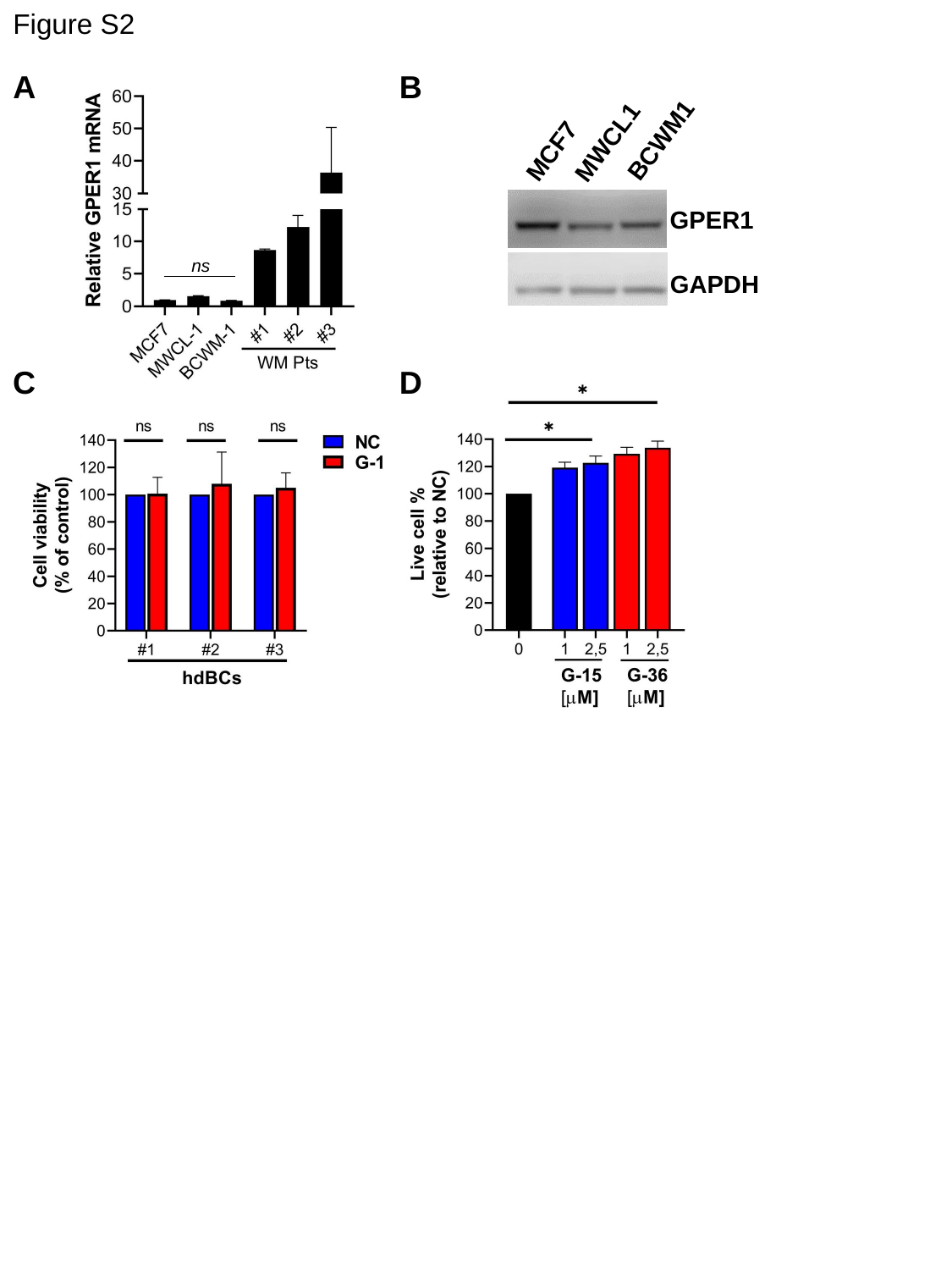

Figure S2
A
B
BCWM1
MWCL1
MCF7
GPER1
ns
GAPDH
C
D

## Slide 3
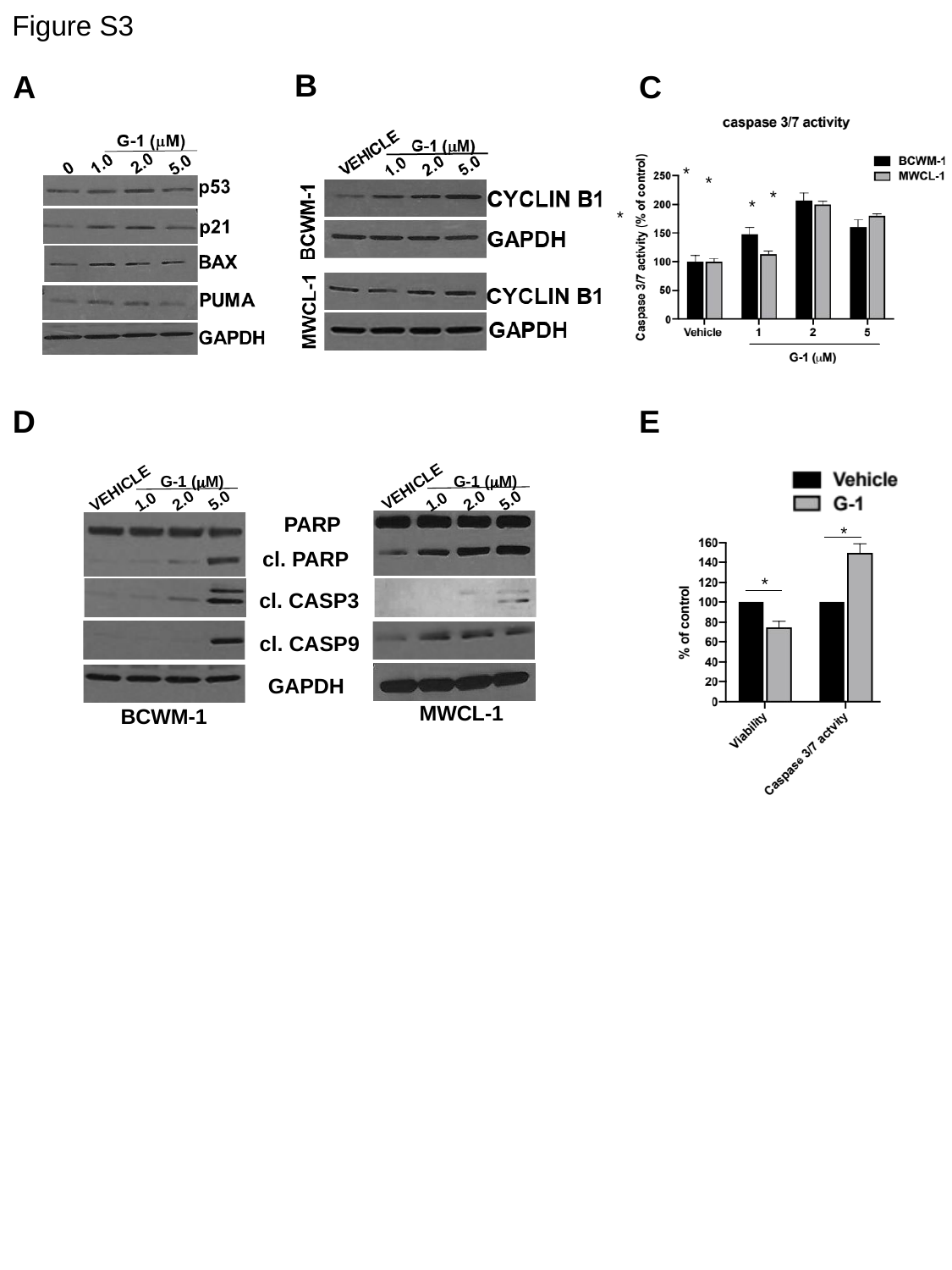

Figure S3
B
A
C
*
*
*
*
*
D
E
G-1 (mM)
G-1 (mM)
VEHICLE
VEHICLE
2.0
5.0
2.0
5.0
1.0
1.0
PARP
cl. PARP
cl. CASP3
cl. CASP9
GAPDH
MWCL-1
BCWM-1
*
*
