# Supplementary material for: Therapeutic activation of G protein-coupled estrogen receptor 1 in Waldenström Macroglobulinemia
Source: Exp Hematol Oncol. 2022 Sep 12;11:54. doi: 10.1186/s40164-022-00305-x (PMC9469525; doi:10.1186/s40164-022-00305-x)
Supplement: Supplementary file 2 — Additional file 2. Methods. [file 40164_2022_305_MOESM2_ESM.docx]

**METHODS**

**Cell cultures**

WM cell lines (BCMW-1 and MWCL-1) were cultured at 37°C in RPMI-1640 medium containing 10% FBS (GIBCO, Life Technologies), 2 mmol/L L-glutamine, 100 U/mL penicillin, and 100 mg/mL streptomycin (GIBCO, Life Technologies). Bone marrow mononuclear cells and primary CD19+ cells from bone marrow aspirates of WM patients were isolated using Ficoll–Hypaque density gradient sedimentation. Primary WM cells were obtained from bone marrow aspirates of patients using CD19 microbead selection (Miltenyi Biotec), resulting in more than 90% purity, as confirmed by flow cytometric analysis. Residual CD19^–^ bone marrow mononuclear cells were cultured in DMEM with 20% FCS for 3 to 6 weeks to generate bone marrow stromal cells (BMSC). Peripheral blood mononuclear cells (PBMCs) were obtained from healthy subjects by Ficoll–Hypaque density gradient sedimentation, and subsequently, CD19 selection was performed. Approval for these studies was obtained from the Dana-Farber Cancer Institute's Institutional Review Board. Informed consent was obtained from all patients and healthy volunteers according to the Declaration of Helsinki protocol. Cells were periodically tested to exclude mycoplasma contamination. Cells were STR (short tandem repeats) authenticated.

**Drugs and oligonucleotides**

Silencer Select siRNA for GPER (siGPER, cat#129900, HSS107126, HSS107127) and for GAPDH (siNC, cat#12935140) were purchased from Thermo Fisher Scientific. G-1 was purchased from Tocris, dissolved in dimethyl sulphoxide (DMSO) at 10 mmol/L, and stored at 80°C for *in vitro* study. Bortezomib was purchased from Selleck.

**Cell viability, apoptosis, cell cycle, and colony forming assays**

A Cell Titer Glo (CTG) assay (Promega) was used to assess cell viability. An Annexin V/7-Aminoactinomycin (7-AAD) flow cytometry assay (BD Biosciences, San Jose, CA, USA) was used to assess apoptosis and cell viability. The staining was performed according to the manufacturer’s instructions. To assess the effect of G-1 on cell-cycle distribution, FACS analysis was performed on MM cells after staining with Propidium Iodide (PI). At each time point, cells were collected, washed twice with phosphate-buffered saline (PBS) and fixed in cold 70% ethanol at 20°C. Before FACS analysis, cells were washed with PBS and stained in 50 μg/ml PI, 100 μg/ml RNase, and 0.05% Nonidet P-40 for 1 h at room temperature in the dark. Cell cycle profiles were determined using FCS Express 6 software. Results are expressed as histogram bars reporting the percentage of each phase. For the colony-forming assay, 200 BCWM-1 cells treated with DMSO or G-1 were plated in triplicate in 1 ml of mixture composed of 1.1% methylcellulose (MethoCultTM STEMCELL Technologies) in RPMI-1640 + 10% FBS. Crystal violet-stained colonies were scored after 2 weeks under an inverted microscope (Leica DM IL LED) at 5× magnification. A Human Apoptosis Array (cat 893900, R&D Systems) was used to assess the protein expression of apoptotic proteins in G-1 treated BCWM-1 cells, according to the manufacturer’s instructions.

**P53 Reporter Assay**

WM cells were transfected with the p53 reporter using the Cignal p53 Response Reporter kit (CCS-004L, Qiagen). Firefly and Renilla luciferase activities were evaluated consecutively using the dual-luciferase assay kit (Promega).

**TEM**

After treatment with G-1, BCWM-1 cell pellets were gently washed with PBS and fixed in 3% glutaraldehyde solution in 0.1 M phosphate buffer (pH 7.4) for 2 hr at 4°C. After osmium tetroxide post-fixation and buffer washes, samples were dehydrated in a graded acetone series and then embedded into epoxy resin. Ultrathin sections (60–90 nm in thickness) were cut with a diamond knife, mounted on copper grids (G300 Cu), and imaged using a Jeol JEM 1400-Plus electron microscope operating at 80 kV.

**Gene Expression Profiling after G-1 treatment.**

WM cells were treated with G-1 or DMSO for 24 hours. RNA was extracted using an RNeasy Mini kit (Qiagen, Hilden, Germany). A total of 300 ng of RNA was used as starting material for preparing the hybridization target by using the GeneChip WT PLUS Reagent Kit (Affymetrix Inc., Santa Clara, CA, USA). The integrity, quality, and quantity of tRNA were assessed using the Agilent Bioanalyzer 2100 (Agilent Technologies, Santa Clara, CA, USA) and NanoDrop 1000 Spectrophotometer (Thermo Scientific, Wilmington, DE). The amplification of cRNA, the clean-up, and the fragmentation were performed according to Affymetrix’s instructions. Microarray data were generated by the GeneChip Human Transcriptome 1.0 Array (Affymetrix Inc.). Arrays were scanned with an Affymetrix GeneChip Scanner 3000. Raw data produced by the Affymetrix Platform (i.e., CEL files) were first processed using the Affymetrix Expression Console (EC). The pre-processing phase was performed according to Affymetrix guidelines and micro-CS software. Raw data were normalized using the probe logarithmic intensity error (PLIER) algorithm coupled to quantile normalization. Annotation of data was also performed using the Affymetrix Provided Libraries and EC version 1.4.1. Differential expression was assessed using a linear model method. *P*-values were adjusted for multiple testing using the Benjamini and Hochberg method. Results were considered to be significant at an adjusted *P*<0.05. Data are available through GEO accession number GSE207488. Functional characterization was performed using the GSEA software v.2.2.1 and the gene sets from the Hallmarks, Kegg, and Reactome collections.

**Gene expression analyses of WM patients and healthy donors.**

WM and healthy donor RNASeq data was obtained from obtained from previously diagnosed patients and healthy volunteers as previously described^1,2^. The data was realigned GRCh37 using Gencode version 24 annotation using two-pass STAR^3^ and quantified with Salmon^4^.

The log10 transformed datasets GSE9656 and GSE61597 were downloaded from NCBI’s gene expression omnibus (GEO) database and were used as validation datasets.

**qRT-PCR analysis of gene expression**

RNA extraction, reverse transcription (RT), and quantitative real-time amplification (qRT-PCR) were performed as previously described^5^. Briefly, total RNA was extracted from cells with TRIzol Reagent (Thermo Fisher Scientific), according to the manufacturer’s instructions. The integrity of total RNA was verified by Nanodrop (Celbio Nanodrop Spectrophotometer nd-1000). cDNA was obtained through the High-Capacity cDNA Reverse Transcription Kit (Thermo Fisher Scientific) and then used as a template to quantify GPER1 (Hs01922715_s1) and GAPDH (Hs03929097_g1). Comparative real-time polymerase chain-reaction (RT-PCR) was performed in triplicate, including no-template controls. Relative expression was calculated using the comparative cross threshold (Ct) method.

**Antibodies and immunoblotting**

Protein extraction and western blot analysis were performed as previously described. Briefly, cells were lysed in 1x RIPA buffer (Cell Signaling Technology) supplemented with Halt Protease Inhibitor Single-Use cocktail (100X, Thermo Scientific). Whole-cell lysates (~20 μg per lane) were separated using 4–12% Novex Bis-Tris SDS-acrylamide gels (Invitrogen) and were electro-transferred onto Nitrocellulose membranes (Bio-Rad). Extraction of nuclear proteins was performed using the NE-PER Nuclear and Cytoplasmic Extraction Reagents (Thermo Fisher, #78833), according to the manufacturer’s instructions. After electrophoresis, the nitrocellulose membranes were blocked and probed overnight with primary antibodies at 4°C, then the membranes were washed 3 times in PBS-Tween and then incubated with a secondary antibody conjugated with horseradish peroxidase for 2 hours at room temperature. Chemiluminescence was detected using the Western Blotting Luminol Reagent (sc-2048, Santa Cruz, Dallas, TX, USA).

Primary antibodies: GPER1 (ab39742) was purchased from Abcam (Cambridge, UK). p21 (#2947), BAX (#5023), PUMA (#98672), and cleaved CASP-3 (#9661) were purchased from Cell Signaling Technology (Danvers, MA). TP53 (sc-126), GAPDH (sc-25778), and β-actin (ab96682) antibodies were purchased from Santa Cruz Biotechnology (Dallas, TX, USA).

Secondary antibodies: Anti-rabbit IgG, HRP-linked antibody (#7074), anti-mouse IgG, and HRP-linked antibody (#7076) were purchased from Cell Signaling Biotechnology (Danvers, MA).

**Immunohistochemistry**

Sections of lymph nodes from patients diagnosed with Waldenstrom macroglobulinemia at the IRCCS AOU San Martino-IST were stained with anti-GPER antibody ([ab154069](https://www.abcam.com/g-protein-coupled-receptor-30-antibody-c-terminal-ab154069.html), Abcam). Immunohistochemistry (IHC) was performed using the Ventana BenchMark XT automated immunostainer. Tissue sections were deparaffinized and rehydrated. After antigen retrieval, sections were incubated with primary antibodies at a dilution of 1:200, and 3,30-diaminobenzidine (DAB) was used as a chromogen. Sections were counterstained with May–Grunwald–Giemsa. For immunohistochemistry staining of tumor xenograft, slices (2 μm size) were deparaffinized and pre-treated with the Epitope Retrieval Solution 2 (EDTA-buffer pH 8.8) at 98°C for 20 min. After the washing steps, peroxidase blocking was carried out for 10 min using the Bond Polymer. All procedures were performed using the Benchmark XT-Automated Immunohistochemistry instrument (Ventana Medical Systems, Oro Valley, AZ, USA). Tissues were again washed and then incubated with the primary antibody directed against caspase-3 (Novocastra, clone JHM62; 1:500), Ki67 (Dako, clone: MIB-1; 1:150) or p53 (DO-7 mAb #48818, Cell Signaling); subsequently, tissues were incubated with polymer for 10 min and developed with DAB-Chromogen for 10 min. Slides were counterstained with hematoxylin for 12 min. Experiments were repeated at least three times.

**Murine xenograft model of human WM**

CB17-SCID mice (28–35 days old) were purchased from Charles River Laboratories. All animal studies were conducted according to protocols approved by the Animal Ethics Committee of the Dana-Farber Cancer Institute. Mice were irradiated (200 cGy) and then inoculated subcutaneously in the right flank with 5 x 10^6^ BCMW.1 cells in 100 μL RPMI-1640. Following detection of tumor (~3 weeks after the injection), mice (n =5/ group) were treated intraperitoneally with G-1 (1 mg/kg body weight) daily for 4 days in a week (with 3 days off), repeated for 3 weeks. The control group received the carrier alone on the same schedule as the combination group. Caliper measurements of the longest perpendicular tumor diameters were performed twice a week to estimate the tumor volume using the following formula: length × width × 0.5. Animals were sacrificed when tumors reached 1 cm^3^ or in the event of a major compromise in their quality of life.

**Synergism quantification**

Drug combination studies and their synergy quantification followed the Chou-Talalay method^6^. Combination indexes (CI) were calculated by CalcuSyn (BIOSOFT, Cambridge, UK).

**Statistical analysis**

Statistical significance of differences observed between drug-treated versus control (in both *in vitro* and *in vivo* experiments) was determined by Student’s t-test. Differences were considered significant when p < 0.05. Tumor growth inhibition and Kaplan–Meier survival analysis were determined using GraphPad Prism analysis software. Drug interactions were analyzed by isobologram analysis using the CalcuSyn Version 2.0 software program (Biosoft). A combination index (CI) < 1.0 indicates synergism, CI = 1 indicates an additive effect, and CI > 1 indicates no significant combination effect (32).

1. Hunter ZR, Xu L, Yang G, et al. Transcriptome sequencing reveals a profile that corresponds to genomic variants in Waldenstrom macroglobulinemia. *Blood*. 2016;128(6):827-838.

2. Hunter ZR, Xu L, Tsakmaklis N, et al. Insights into the genomic landscape of MYD88 wild-type Waldenstrom macroglobulinemia. *Blood Adv*. 2018;2(21):2937-2946.

3. Dobin A, Davis CA, Schlesinger F, et al. STAR: ultrafast universal RNA-seq aligner. *Bioinformatics*. 2013;29(1):15-21.

4. Patro R, Duggal G, Love MI, Irizarry RA, Kingsford C. Salmon provides fast and bias-aware quantification of transcript expression. *Nat Methods*. 2017;14(4):417-419.

5. Morelli E, Biamonte L, Federico C, et al. Therapeutic vulnerability of multiple myeloma to MIR17PTi, a first-in-class inhibitor of pri-miR-17-92. *Blood*. 2018;132(10):1050-1063.

6. Chou TC. Drug combination studies and their synergy quantification using the Chou-Talalay method. *Cancer Res*. 2010;70(2):440-446.
